# Habitat Selectivity and Reliance on Live Corals for Indo-Pacific Hawkfishes (Family: Cirrhitidae)

**DOI:** 10.1371/journal.pone.0138136

**Published:** 2015-11-03

**Authors:** Darren J. Coker, Andrew S. Hoey, Shaun K. Wilson, Martial Depczynski, Nicholas A. J. Graham, Jean-Paul A. Hobbs, Thomas H. Holmes, Morgan S. Pratchett

**Affiliations:** 1 ARC Centre of Excellence for Coral Reef Studies, James Cook University, Townsville, Australia; 2 Red Sea Research Center, Division of Biological and Environmental Science and Engineering, King Abdullah University of Science and Technology, Thuwal, Saudi Arabia; 3 Oceans Institute, The University of Western Australia, Crawley, Australia; 4 Marine Science Program, Department of Parks and Wildlife, Perth, Australia; 5 Australian Institute of Marine Science, Oceans Institute, The University of Western Australia, Crawley, Australia; 6 Lancaster Environment Centre, Lancaster University, Lancaster, United Kingdom; 7 Department of Environment and Agriculture, Curtin University, Perth, Australia; Biodiversity Insitute of Ontario - University of Guelph, CANADA

## Abstract

Hawkfishes (family: Cirrhitidae) are small conspicuous reef predators that commonly perch on, or shelter within, the branches of coral colonies. This study examined habitat associations of hawkfishes, and explicitly tested whether hawkfishes associate with specific types of live coral. Live coral use and habitat selectivity of hawkfishes was explored at six locations from Chagos in the central Indian Ocean extending east to Fiji in the Pacific Ocean. A total of 529 hawkfishes from seven species were recorded across all locations with 63% of individuals observed perching on, or sheltering within, live coral colonies. Five species (all except *Cirrhitus pinnulatus* and *Cirrhitichthys oxycephalus*) associated with live coral habitats. *Cirrhitichthys falco* selected for species of *Pocillopora* while *Paracirrhites arcatus* and *P*. *forsteri* selected for both *Pocillopora* and *Acropora*, revealing that these habitats are used disproportionately more than expected based on the local cover of these coral genera. Habitat selection was consistent across geographic locations, and species of *Pocillopora* were the most frequently used and most consistently selected even though this coral genus never comprised more than 6% of the total coral cover at any of the locations. Across locations, *Paracirrhites arcatus* and *P*. *forsteri* were the most abundant species and variation in their abundance corresponded with local patterns of live coral cover and abundance of Pocilloporid corals, respectively. These findings demonstrate the link between small predatory fishes and live coral habitats adding to the growing body of literature highlighting that live corals (especially erect branching corals) are critically important for sustaining high abundance and diversity of fishes on coral reefs.

## Introduction

Strong microhabitat associations can have a major bearing on the distribution, abundance and fitness of populations and/or species through the provision of resources (e.g., food, breeding sites), and by mediating exposure to predators and competitors [1,2,3,4]. Species with a strong reliance on a limited set of microhabitat types (i.e. habitat specialists) often have lower abundances than their generalist counterparts [5,6,7,8]. However, specialist species may outcompete and exclude generalists from using preferred microhabitats [9,10]. Currently, one of the most pressing concerns in today’s rapidly changing environments is that highly specialised species may be particularly prone to significant and widespread habitat degradation [5,11,12].

For many specialist and generalist small coral reef fishes (including juveniles of larger fishes) and motile invertebrates, live corals provide an essential microhabitat offering food, shelter from predation, or breeding sites [13,14,15,16,17]. In addition, studies have shown that the abundance of coral reef fishes is strongly and positively correlated with live coral cover [18,19,20]. Even more importantly, the individual abundance of many reef fishes has been shown to decline in accordance with sudden or pronounced coral loss [21,22,23].

Branching corals, such as most species within the scleractinian families (e.g., Acroporidae and Pocilloporidae), are key habitat-forming species on tropical reefs, yet they are highly susceptible to a wide range of natural and anthropogenic disturbances including climate-induced bleaching, outbreaks of coral feeding starfish, disease, and severe tropical storms [24,25,26,27,28]. The increasing incidence of these disturbances is leading to sustained declines in live coral and loss of habitat structure at many locations [29,27]. How reef fishes are affected by, and respond to, sustained declines in the availability of coral microhabitats depends on their degree of habitat-specialisation and reliance on coral [21,22]. However, there is still a lack of information on what coral species fishes prefer and if they have the ability to utilise alternative habitats following the loss of preferred coral habitats. Determining the specifics of reef fish habitat requirements is important in understanding what elements of habitat are essential for ecosystem function and resilience.

Previous research on habitat specialisation in coral-dwelling fishes has generally focussed on newly settled or small benthic feeding fishes (e.g., [15,30,31]). There is however, a significant knowledge gap in the level of habitat specialisation for other fish groups, especially predators. Hawkfishes are small demersal reef predators, with 33 species distributed across the tropical Western and Eastern Atlantic, Indian and Pacific Ocean [32]. Hawkfishes are generally observed perched on raised substrata, often corals, which serve the purposes of protection against larger predators, a vantage point for hunting small fish and crustaceans [32,33], and/or for courting and spawning [34]. Despite this apparent association with live coral there is a lack of information on the specific microhabitat preferences for most species of hawkfish. The majority of research on habitat use in hawkfishes has focussed on a single species, the arc-eye hawkfish *Paracirrhites arcatus* (e.g., [35,36]), while reproductive behaviour has been extensively studied for another species, *Cirrhitichthys falco* (e.g., [37,38,39]). The objective of this study, therefore, is to examine the patterns of habitat use by hawkfishes (family: Cirrhitidae) and to determine the degree of reliance of individual species on live coral. Specifically, the aims of the study are to i) quantify the distribution and abundance of hawkfish assemblages on shallow coral reefs across a broad geographic range, from Chagos in the Central Indian Ocean to Fiji in the Central Pacific Ocean, ii) determine the affiliation and habitat selectivity of individual hawkfish species to live coral/habitat types, and iii) to determine whether habitat associations are consistent across the geographic scale of the study.

## Materials and Methods

### Survey sites

**Fig 1:**
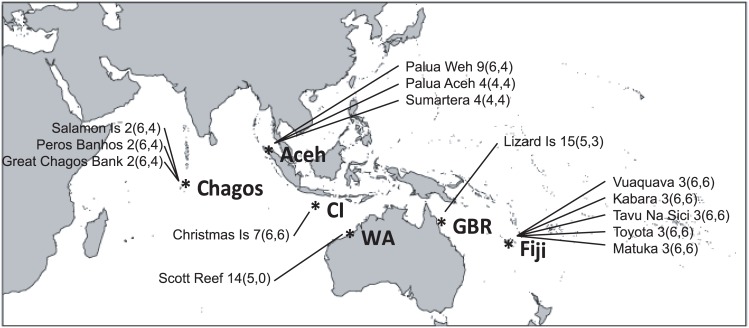
Reefs and number of sites sampled within each location. Key: number of sites (number of abundance transects, number of substrate transects). Location abbreviations: CI = Christmas Island, WA = Western Australia, and GBR = Great Barrier Reef.

Surveys of abundance and habitat use for hawkfishes (family: Cirrhitidae) were recorded at six geographically distinct locations across both the Indian (Chagos, Aceh, Christmas Island (CI), and Western Australia (WA)) and Pacific (Great Barrier Reef (GBR) and Fiji) Oceans. The distances between these locations were greater than 2,500 km and spanned a total of 11,000 km of geographic distance. A hierarchical sampling design was used to record abundance and habitat association with 3–6 replicate transects within each of 2–15 sites at each location (see Fig 1 for details). Data used in this study was derived from independent studies conducted in the six different locations, such that there were slight variations in the sampling intensity and units used. However, all surveys were conducted at a water depth of 3–6 m, by divers using SCUBA between 08:00 and 17:00 hours during the Austral summer months. No specific permissions were required for these locations/activities. All fieldwork and data collection was observational and non-extractive. The field studies did not involve endangered or protected species.

### Habitat availability

Substratum composition was recorded to document the availability of benthic groups. Habitat data was derived from replicate 20 m line intercept transects from the GBR and 30 m point intercept transects (PIT) from Aceh, Christmas Island, Fiji, and 50 m from Chagos (see Fig 1 for replicate details). The substratum was recorded under uniform points (0.5 m intervals) for PIT. Where possible, live coral was recorded to species level, but subsequently pooled to the following nine habitat categories; *Acropora*, *Pocillopora*, *Porites*, *Stylophora*, other hard coral, dead coral (intact but dead coral colonies), pavement, non-coral and soft coral. This technique provided an estimate of the habitats available to fish at each location. Variation in proportional cover of different habitat categories was analysed using a permutational multivariate analysis of variance [40] in R with the package *vegan* [41].

### Community patterns in hawkfishes

To determine the abundance of hawkfishes, visual belt transects were conducted at each location. Transects ranged from 10 x 1m in the GBR to 30 x 1 m in Chagos, Aceh, Christmas Island, and Fiji and 50 x 2 m in WA (see Fig. 1 for replicate details). Variation in the transect dimensions (mainly width) are not expected to greatly affect estimates of abundance (*sensu* [42]), and are within the range of dimensions (especially the width of transects) that have been used previously to survey hawkfishes [35]. A total of 19 km^2^ was surveyed across the six locations. To standardise differences in sampling area, abundance was converted to density (number of fish per m^2^) for each transect and compared among locations using ANOVA. To explore variation in hawkfish assemblage structure across and within geographic locations, a non-metric multi-dimensional scaling (MDS) analysis based on Bray-Curtis dissimilarity was used. The BIOENV routine was then used to explore what habitat variables best correlate (Spearman rank correlation) to the observed hawkfish assemblages [43]. Analyses were conducted in R using the package *vegan*.

To further identify habitat variables and parameters that may predict the observed abundance patterns among hawkfishes, distance-based linear models (DISTLM) were conducted on the two most abundant species, *Paracirrhites arcatus* and *P*. *forsteri*. The analyses were based on the abundance of hawkfishes and the benthic substratum recorded along each transect, with the dependent variable being the abundance of each hawkfish species and the predictor variables the cover of *Acropora*, Pocilloporidae, *Porites*, other hard coral (OHC), and dead coral (DC) which included dead coral, rubble, and pavement. All combinations of predictor variables were considered in the models, with the best combination of variables producing the lowest Akaike Information Criteria (AICc) with the least number of variables. In addition, all combinations within two AICc units of this model were retained [44]. Weighted AICc values were summed across all possible models to explore the relative importance of each variable. Analyses were performed in PRIMER and PERMANOVA+ V6 [45].

### Habitat association

Habitat association of hawkfishes across the six locations were conducted simultaneously during the surveys to document patterns of microhabitat use. For each hawkfish encountered during the visual surveys, the substratum on which individuals were perching on or sheltering within, when first observed, was recorded. Substratum categories were the same as those used for the benthic surveys. Habitat specialisation for each hawkfish species was then determined using Smith’s measure of niche breadth (*FT*). *FT* is a standardised measure of specialisation and niche breadth that evaluates the range of resources used by a species within a location. Calculated values for each species and location are between a minimum of 0 (habitat specialist) and a maximum of 1 (habitat generalist). This measure takes into account resource availability and is much less sensitive to the selectivity of rare resources over other measures [46]. Niche breadth (*FT*) was calculated with 95% confidence intervals [46] for each of the nine habitat categories at each site using:

$$FT=\sum \left(\sqrt{p_{j}a_{j}}\right)$$

where *p*_*j*_ is the proportion of individuals using habitat category *j* and *a*_*j*_ the proportion of total habitats comprised by resource *j*. Confidence intervals (95%) were calculated using the arcsine transformation:

$$\text{sin}[x\pm \frac{1.96}{2\sqrt{Y}}]$$

where *x* = Arcsin (*FT*) and *Y* = Total number of individuals studies = ∑N_*j*_.

Niche breadth was calculated for each species in each location, providing comparisons of overall levels of specialisation in habitat use among locations and species. Species without sufficient records of habitat association at each location were omitted from the analysis.

### Size specific habitat relationships

For individual hawkfishes associating with live coral colonies in Christmas Island and Fiji, the size of individual fish (TL, nearest cm) and size of their host colony (maximum diameter: 0–20, 20–30, 30–40, > 50 cm) was estimated to examine if there was a relationship between fish size and coral colony size. Separate regression analyses were conducted for *P*. *arcatus* and *P*. *forsteri* (pooled across the two locations), and *P*. *hemistictus* from Christmas Island. To determine if fish coral relations differed between locations, the analysis was run with and without location as a random factor. The two models were compared (likelihood ratio test) and location was found to be non significant for both *P*. *arcatus* (χ^2^ = 0.009, P = 0.94) and *P*. *forsteri* (χ^2^ = 0.000, P = 1.00). Results were therefore reported without location as a random factor in the model (package *lme4* in R). Size-specific habitat use (*Acropora*, Pocilloporidae, and other live hard corals) was tested for these three hawkfish species using ANOVA.

### Habitat selectivity

Resource selectivity ratios were calculated to investigate if hawkfishes associate with any specific habitats disproportional to availability. The resource selectivity ratios were based on the nine benthic categories identified above as not all coral species were present at all sites and locations, and pooling coral data to genus facilitated greater comparison of habitat selection among locations. Resource selectivity functions were calculated for each hawkfish species, at the reef level, for each of the nine habitat categories following [47] Model Design I, Sampling Protocol A, which allowed for random sampling of used resource units and available resource units at the population level. Selection ratios (*w*_*i*_) were calculated with the formula:

$$w_{i}=o_{i}/Pr_{\left(i\right)}$$

where *o*_*i*_ is the proportion of all habitat occupied by a species of hawkfish in which the habitat is *i* and *Pr*_(i)_ is the proportion of total available habitat that is *i* [47]. Bonferroni corrected 95% confidence intervals (CI) were calculated for each selection ratio to account for multiple comparisons using the formula:

$${\text{Z}}_{\text{a}/2\text{k}}\sqrt{\left[oi\left(1-oi\right)/\left(U+\mathrm{Pr}\left(i\right)2\right)\right]}$$

where Z_a/2k_ is the critical value of the standard normal distribution corresponding to an upper tail area of _a/2k_, *a* = 0.05, *k* = the total number of habitats use, and *U+* is the total number of habitats of all categories used by that species of hawkfish. A significant positive use of habitat was indicated if selection indices (± 95% CI) were above the value of one, while a value (for each species within a location) around one was regarded as habitat used in equal portions to its availability, and a value below one indicates disproportionally low use of a habitat [47]. Selection ratios were only calculated for sites in which more than five individuals were recorded per hawkfish species.

## Results

### Habitat availability

**Fig 2:**
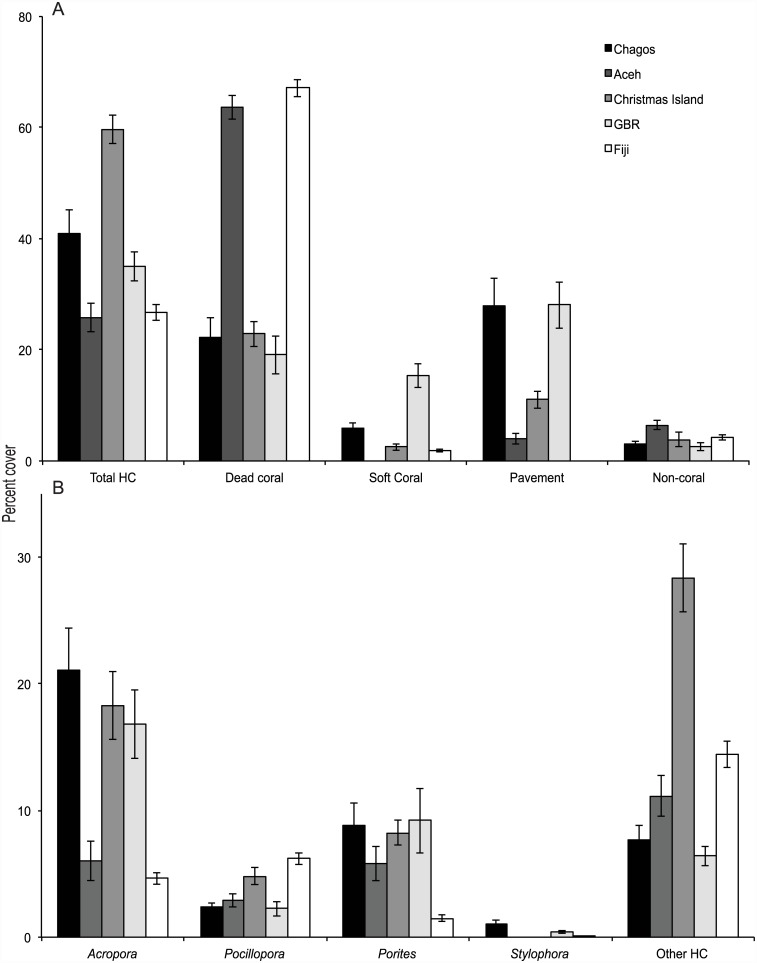
Mean percentage cover (± SE) of habitat categories at six study locations. (A) Three non-live scleractinian categories, soft coral, and pooled live hard coral (Total HC). (B) Four common coral genera and pooled other hard live corals (Other HC). Means for each location calculated from transects across all reefs.

The cover of live coral (hard and soft coral combined) differed significantly between locations (F_5,9_ = 6.34, P<0.001) and among reefs within locations (F_9,40_ = 10.37, P<0.001). Hard coral cover was greatest at Christmas Island (59.0% ± 2.6 SE), lowest in Aceh and Fiji (26% ± 2.6 and 27% ± 1.5, respectively) and intermediate in Chagos and Australia’s Great Barrier Reef (35–40%, Fig 2A). Dead coral cover ranged from 19 to 67%, contributing a significant proportion of the substrate in some locations. Coral assemblages tended to be dominated by *Acropora* species and varied in cover from 5% in Fiji up to 22% in Chagos (Fig 2B). *Pocillopora* cover was relatively low across all locations ranging from 2% cover in Aceh to 6% in Fiji. The other major genera of hard corals, *Porites* spp. ranged between 1% cover in Fiji up to 9% in Chagos. Other hard corals made up the remaining component of the live coral cover and was particularly high in Christmas Island (28%).

### Community patterns in hawkfishes

**Fig 3:**
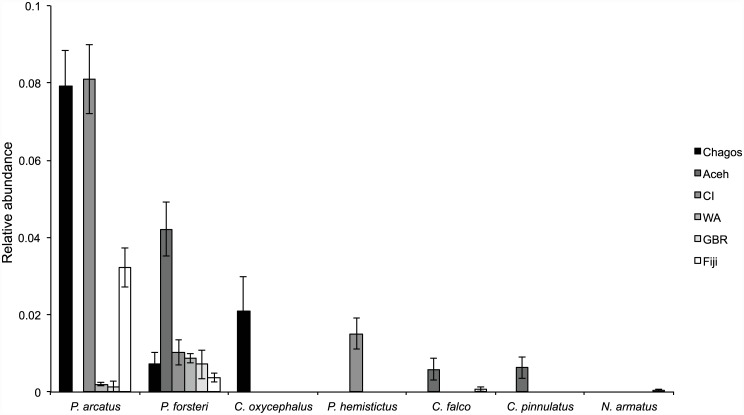
Mean (per m^2^ ± SE) relative abundance of seven hawkfish species at six locations.

A total of 526 individual hawkfishes from seven different species were recorded across the six locations. Overall, *Paracirrhites arcatus* (268 individuals) and *P*. *forsteri* (185 individuals) were the most abundant, while *Cirrhitichthys oxycephalus*, *P*. *hemistictus*, *C*. *falco* and *Cirrhitus pinnulatus* were much less common (25, 18, 16, and 13 individuals, respectively). Only one *Neocirrhites armatus* individual was observed in Vauaqava (Fiji) and no species were observed from the remaining 17 species known to exist within the study area, suggesting they are either rare, extremely cryptic or occur in locations/habitats that were not surveyed (e.g., sheltered and deeper sites, different reef zones). *Paracirrhites arcatus* was the most abundant species overall, but displayed marked variation among sites, from 0–0.08 fish per m^2^ at Aceh and Christmas Island, respectively (Fig 3). *Paracirrhites forsteri* was the only species recorded on reefs at all six locations and was four times more abundant in Aceh (mean 0.04 fish per m^2^ ± SE 0.01) than the other five locations (range: 0.004–0.009 fish per m^2^). The remaining five species were relatively uncommon, recorded at only one or two of the six locations.

**Fig 4:**
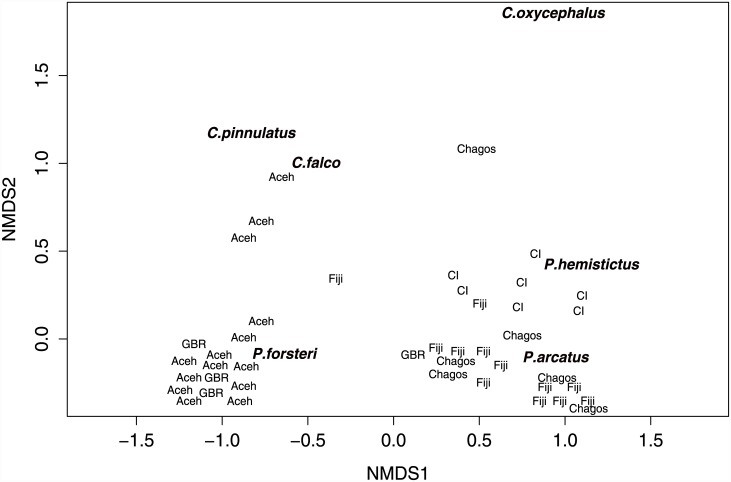
Multidimensional-scaling plot (MDS) of reefs within locations based on hawkfish community composition. Each location point represents a reef within the location. Location abbreviations: CI = Christmas Island, and GBR = Great Barrier Reef. Position of hawkfish species represents correlations between fish assemblages and reefs based on habitat variables. Plot stress = 0.043.

Composition of hawkfish assemblages varied among the six locations (PERMANOVA F_4,34_ = 34.62, P = <0.001). The GBR and Aceh hawkfish assemblages were separated from those of the Fiji, Chagos and Christmas Island assemblages along the first dimension of the MDS (Fig 4). The combination of habitat variables that best correlates with hawkfish community patterns across locations were Pocilloporidae and dead coral (Spearman rank correlation: 0.124). Aceh and GBR assemblages were characterised by a relatively high abundance of *P*. *forsteri*, Christmas Island by *P*. *hemistictus*, and Fiji by *P*. *arcatus* (Fig 4).

At the species level, 23% of the variation in *P*. *arcatus* abundance was explained best by the combined proportional cover of all live coral groups, and revealed no support for dead coral substrata (Table 1). In addition, summed AICc weights further support the importance of live corals in predicting abundance (Table 2). Combinations of all substrata groups tested explained weak relationships between habitat and the abundance of *P*. *forsteri* (Table 1), although summed AICc weights showed strong support for Pocilloporidae corals in explaining abundance patterns along with high variation among locations (Table 2). Relationships between abundance and available resource habitats could not be confidently tested for the other five species of hawkfish due to their low occurrences.

**Table 1:** Best models for predicting the influence of available substrata on the abundance of *Paracirrhites arcatus* and *P*. *forsteri*.

Model	K	AICc	ΔAICc	AIC wt	*R2*
***P*. *arcatus***					
*Acropora*, OHC, Pocilloporidae, *Porites*, Location	7	1384.6	0	0.342	0.226
*Acropora*, OHC, Pocilloporidae, *Porites*	6	1385.2	0.6	0.254	0.218
***P*. *forsteri***					
Pociiloporidae, Location	4	1298	0	0.169	0.137
*Acropora*, Pocilloporidae, DC, Location	6	1298	0	0.169	0.151
Pocilloporidae, OHC, Location	5	1298.6	0.6	0.125	0.142
*Acropora*, Pocilloporidae, Location	5	1298.7	0.7	0.119	0.142
*Acropora*, Pocilloporidae, OHC, Location	6	1299.4	1.4	0.084	0.146
Pociiloporidae, DC, Location	5	1299.7	1.7	0.072	0.139
Pociiloporidae, *Porites*, Location	5	1299.7	1.7	0.072	0.139
*Acropora*, Pocilloporidae, *Porites*, DC, Location	7	1299.9	1.9	0.065	0.151

Distance–based linear models (DISTLM) for *P*. *arcatus* and *P*. *forsteri* across all locations. Best models shown are based on Akaike Information Criteria (AICc) values and weighted AIC values (AIC wt) and are within 2 units of the lowest AICc value. Predictor variables included *Acropora*, Pocilloporidae, *Porites*, other hard coral (OHC), and dead coral (DC). All models contained the random factor location.

**Table 2:** Relative importance of each habitat variable on the abundance of *Paracirrhites arcatus* and *P*. *forsteri*.

	*P*. *arcatus*	*P*. *forsteri*
*Acropora*	0.89	0.5
Pocilloporidae	1	1
*Porites*	0.91	0.2
OHC	1	0.33
DC	0.4	0.37
Location	0.56	1

Summed AICc weights (across all models) for abundance of *P*. *arcatus* and *P*. *forsteri* predictor variables. OHC = other hard corals, DC = dead coral.

### Habitat associations

**Fig 5:**
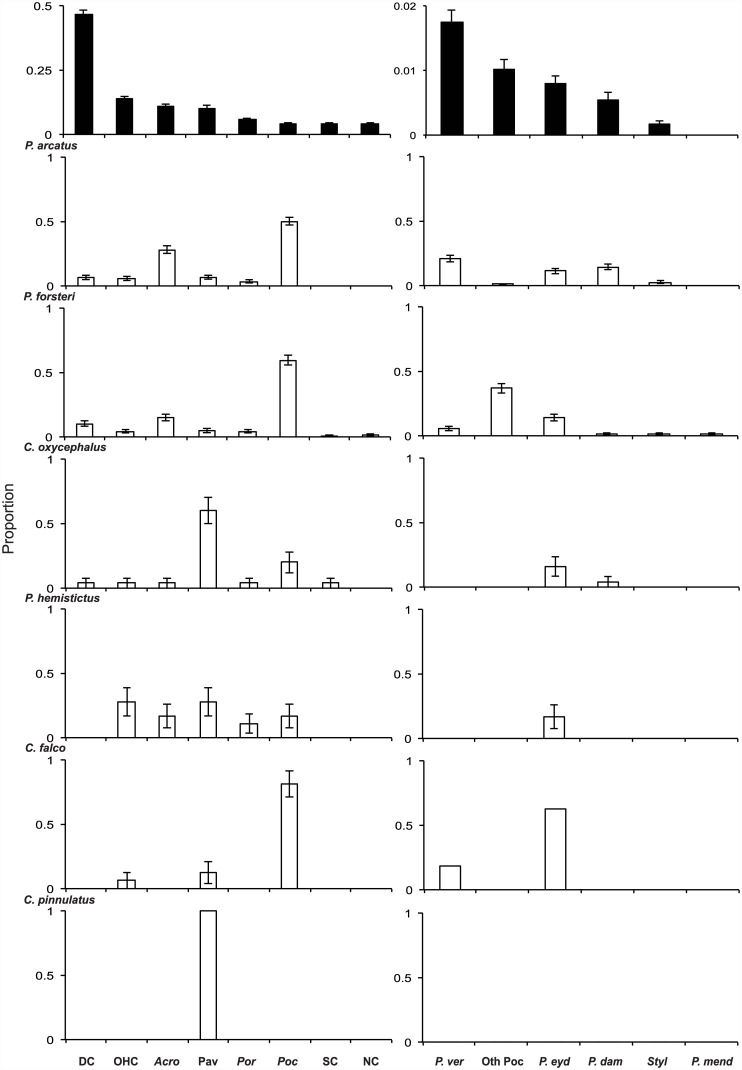
Proportion of available versus used habitat categories for hawkfishes. Proportion of available habitat categories (black bars) across all locations and the proportion of fish that each benthic category was used by each hawkfish species (white bars). Figures on the left display habitat associations for each species across all habitat categories and on the right among different species of Pocilloporidae. Note scale differences for available. Abbreviations: DC = dead coral, OHC = other hard corals, Acro = *Acropora*, Pav = pavement, Por = *Porites*, Poc = Pocilloporidae, SC = soft coral, NC = Non-coral, P.ve r = *Pocillopora verrucosa*, Oth Poc = Other Pocilloporidae, P.eyd = *P*. *eydouxi*, P.dam = *P*. *damicornis*, Styl = *Stylophora*, P.mend = *P*. *meandrina*.

Despite the relative low cover of Pocilloporidae across all locations (max 6%), the use of these corals by hawkfish was relatively high. Overall, 45% of all hawkfish were observed sheltering or perching on Pocilloporidae and 26% on *Acropora* species. A high percentage of *P*. *forsteri*, *P*. *arcatus*, *C*. *falco* and the single individual of *N*. *armatus* were recorded on species of Pocilloporidae regardless of the local availability of these corals (Fig 5). Among species of Pocilloporidae, *P*. *arcatus* was more commonly observed on *Pocillopora verrucosa* (21% ± SE 2.5), *P*. *damicornis* (14% ± SE 2.1), and *P*. *eydouxi* (11% ± SE 1.9) and *C*. *falco* was commonly observed with *P*. *verrucosa* (19% ± SE 10.1). All other species, except *C*. *pinnulatus*, associated with *P*. *eydouxi* (Fig 5).

Niche breadth (*FT*) with 95% confidence intervals (CI) for *P*. *arcatus* varied from 0.39 (± CI 0.01) in Fiji up to 0.65 (± CI 0.01) and 0.82 (± CI 0.01) for Chagos and Christmas Island respectively. For *P*. *forsteri*, niche breadth ranged from 0.43 (± CI 0.17) and 0.48 (± CI 0.01) for GBR and Aceh respectively to 0.79 (± CI 0.08) and 0.81 (± CI 0.01) for Christmas Island and WA respectively showing some variability between locations within species but an overall low specialisation of habitat use (i.e., values close to 1). Habitat specialisation was also low for *P*. *hemistictus* 0.82 (± CI 0.06) and *C*. *oxycephalus* 0.53 (± CI 0.07) but high for *C*. *pinnulatus* 0.20 (± 0.15) in the observed locations (Christmas Island, Chagos and Aceh respectively).

### Size specific habitat relationships

There were significant differences among the mean observed size (TL) of the three most common hawkfish species (F_2,2_ = 50.26, P<0.001) ranging from 7.5 cm (± SE 0.23) for *P*. *arcatus*, to 9.3 cm (± SE 0.66) for *P*. *forsteri* to 15.5 cm (± SE 0.32) for *P*. *hemistictus* (S1 Fig). However, there was no evidence of size-specific habitat use for these three abundant species (F_2,2_ = 0.85, P = 0.496) with similar sized individuals within each hawkfish species associating with a range of habitat types, including species of *Pocillopora*, *Acropora*, and other corals.

There were statistically significant but very weak relationships observed between body size (TL) and coral colony size (max dia.) for *P*.*arcatus* (R^2^ = 0.10, F_1,68_ = 18.87, P<0.01; y = 0.75x+5.89) and *P*. *forsteri* (R^2^ = 0.34, F_1,17_ = 10.20, P = 0.01: y = 2.41x+5.08), however these relationships are likely to be biologically insignificant. The largest hawkfish species *P*. *hemistictus*, did not reveal a relationship (R^2^ = 0.06, F_1,15_ = 0.12, P = 0.73) (S2 Fig). Individual *P*. *arcatus* and *P*. *hemistictus* associated with a wide range of colony sizes (8 to >50 cm mean dia.) regardless of body size. While *P*. *forsteri* revealed a slight positive but non-significant relationship between TL and colony size they were not observed to associate with coral colonies larger than 40 cm max dia. despite their availability. Overall, these species did not show any evidence of size specific habitat relationships with many large individuals of all species found to associate with small colonies.

### Habitat selectivity

Based on the frequency of use relative to the proportional availability of the substrata, habitat selectivity of hawkfishes varied among species and among locations. Four of the six hawkfish species recorded selected live coral habitats in greater proportion than other available habitats (Table 3). The two most widespread species *P*. *arcatus* and *P*. *forsteri* consistently selected for *Pocillopora* and *Acropora* corals. Interestingly, *P*. *arcatus* selected *Pocillopora* corals at almost all reefs where it was observed, the exception being in Chagos, where *Stylophora* corals were relatively more abundant and the preferred coral genera. Similarly, *C*. *falco* selected for *Pocillopora* whilst *P*. *hemistictus* selected other hard corals and pavement habitats, although both these species were only recorded at single locations. In contrast to those species that selected for live corals, both *C*. *pinnulatus* and *C*. *oxycephalus* displayed positive selection for pavement habitats.

**Table 3:** Habitat selectivity of hawkfishes.

	Location	Reef	*Stylophora*	*Pocillopora*	*Acropora*	*Porites*	OHC	DC	Pavement	Non-coral	Soft coral
***P*. *arcatus***
	Chagos	Great Chagos	o	o	+	o	o	o	o	o	o
		Peros Banhos	+	o	o	o	o	o	o	o	o
		Salamon	+	o	o	o	o	o	o	o	o
	Fiji	Matuku		+	+	o	o	o		o	
		Totoya		+	+	+	−	o	−	o	
		Tuvu Na Sici		+	+	o	o	o	−	o	
		Vuaqava		+	+	o	−	o	−	o	
	CI	Ethel Beach		+	o	+	o	o	o	o	
		Flyingfish Cove	o	+	=	o	=	+	−	o	o
		Jacksons Point	o	+	+	=	+	o		o	o
		Ryans Ravine	o	+	+	=	o	o		o	o
		Thomas Point	o	=	+	=	o	o		o	o
		Thundercliff	o	+	=	−	=	−	+	o	o
***P*. *forsteri***
	Aceh	Pulau Aceh		+	o	o	o	o	o	o	
		Pulau Weh		+	+	+	−	−	o	o	o
		Sumatera		+	o	o	o	o	o	o	
	CI	Thundercliff	o	=	=	−	+	o	+	o	o
***C*. *oxycephalus***
	Chagos	Salamon	o	o	o	o	o	o	+	o	o
***P*. *hemistictus***
	CI	Thundercliff	o	o	o	−	+	o	+	o	o
***C*. *falco***
	Aceh	Pulau Weh		+	o	o	o	o	o	o	o
***C*. *pinnulatus***
	Aceh	Pulau Weh		o	o	o	o	o	+	o	o
		Sumatera		o	o	o	o	o	+	o	

‘+’ indicates habitat used significantly more than expected,

‘ = ‘ habitat used in proportion to availability,

‘-‘ habitat used significantly less than expected,

‘o’ habitat not used.

OHC = other hard coral,

DC = dead coral.

## Discussion

Broad geographic surveys of hawkfishes revealed differences in overall abundance and assemblage structure across locations. *Paracirrhites arcatus* was the most abundant of the seven recorded species and was the dominant species in Chagos, Aceh and Fiji. Also relatively abundant, *P*. *forsteri* was the only species recorded across all locations. Variation in abundance among locations and reefs suggest that a multitude of processes (e.g., recruitment, predation, competition) are acting on these species over their geographic range. The majority of species were associated with live coral, with four of the seven species displaying positive selection for live pocilloporid and acroporid coral colonies. At local scales, colonies of *Pocillopora* were consistently selected by *P*. *arcatus*, *P*. *forsteri* and *C*. *falco* and appear to be the favoured habitat despite the relatively low abundance of this coral at most locations (2–6% cover). Accordingly, the availability of live coral colonies explain 15 and 22% of the variation in the abundance of *P*. *arcatus* and *P*. *forsteri* respectively across locations, suggesting that live branching corals play some role in shaping abundance and distribution patterns of hawkfishes.

Similar habitat associations have been reported previously in Hawaii and the lagoons of French Polynesia where *P*. *arcatus* used *Pocillopora* colonies disproportionately to their availability [35,36]. This study shows that this selection is conserved across a wider geographic range for *P*. *arcatus* and also extends to *P*. *forsteri* and *C*. *falco*. In addition, the former two species showed strong selectivity for *Acropora* habitats in over half of the reefs where present. *Acropora* and *Pocillopora* colonies are often large (e.g., *A*. *hyacinthus*, *P*. *eydouxi*) or form expansive thickets (e.g., *A*. *formosa*, *P*. *damicornis*) and are proposed to provide two important functions for hawkfishes. Firstly, this structure provides an important shelter through the availability of a complex structure and deep refuge spaces for small fishes to retreat into when threatened by predators [35]. Secondly, these structures are an important habitat for prey [48,49] and may provide a structure from which to launch an attack [50,51,52]. Small coral-dwelling fishes (e.g., damselfish) and crustaceans are important prey items for hawkfishes [50,53,54] and are known to be abundant within live complex colonies [16,49]. Interestingly, DeMartini [55] reported that approximately 60% of predatory strikes by *P*. *arcatus* were on the substratum rather than the water column, and of these, 81–96% were directed at prey on dead coral and rocks, as opposed to within live coral colonies. This relationship implies corals may be favoured as a shelter or raised platform from which *P*. *arcatus* can launch attacks and defend their territory rather than as a prey source. This feeding strategy may vary among species, but regardless of the mechanisms, these hawkfishes appear to have a clear preference for live *Acropora* and pocilloporid species.

While the majority of hawkfishes were recorded on live coral, *C*. *oxycephalus* and *C*. *pinnulatus* both selected for pavement habitats. Individuals of *C*. *oxycephalus* were observed on all categories of live coral, but the majority were documented on pavement habitats. It appears that this species uses this microhabitat proportionately more for daily activities than nearby live corals, although this may make them more vulnerable to predation. *Cirrhitus pinnulatus* appears to be a habitat specialist, displaying a narrow niche breadth and only documented on pavement microhabitats. This species is large (ca. max 280 mm TL) and has markings and colouration complementing non-coral hues and patterns. These physical characteristics suggest that this species is more suited to non-coral areas of the reef using camouflage and spaces between colonies and within the reef matrix for shelter rather than among coral colony branches. Interesting, *C*. *oxycephalus* and *C*. *pinnulatus* are widespread species but were only documented in Chagos and Aceh respectively. Clearly, further research is needed to elucidate differences in microhabitat requirements and the underlying mechanisms among hawkfish species.

In this study, the majority of hawkfishes were found associating with live coral, and while five of the seven species appeared to preferentially associate with live coral habitat, they do not appear to be obligately dependent on live corals and often associate with alternative, non-preferred habitats. This was supported by a low to medium niche breadth for all but *C*. *pinnulatus* among locations suggesting that these species are habitat generalists but with strong habitat preferences, with species either using these alternative habitats for specific actions or when preferred habitats are scarce or unavailable. Across the study sites, *Pocillopora* only accounted for 2–6% cover. Thus, given the rarity of *Pocillopora*, these hawkfishes may be forced to associate with alternative habitats. The rarity or loss of preferred habitats may have indirect effects on hawkfish fitness if alternative habitats do not fully provide essential resources (e.g., shelter, access to prey, prey capture success). Alternatively, hawkfishes may utilise other microhabitats for different functions within their territory (e.g., territory defence, mate guarding). Hawkfishes are extremely territorial, with estimates of territory size ranging from ca. 3 m^2^ (female *C*. *falco*, [37]) to ca. 150 m^2^ (male *P*. *hemistictus*, [34]). Hawkfishes move around within a defended territory, and documented microhabitats may not offer any specific ecological benefits other than a stepping-stone between important functional habitats. Furthermore, individuals may utilize different colonies during the day (feeding and territory maintenance) versus night (shelter).

Size of coral colonies may also relate to suitability as a shelter or availability of prey, larger hawkfish potentially require larger colonies with wider refuges and more prey than smaller bodied counterparts. Previous research has found that *P*. *arcatus* preferred large, opened-branched *Pocillopora* colonies but may use smaller, more tightly branched colonies when their preferred habitat is rare or absent [35]. The present study found no evidence for ecologically significant relationships between total length (TL) and the type of live coral used, or TL and colony size (max dia.) for *P*. *arcatus*, *P*. *forsteri*, and *P*. *hemistictus*. There was a weak positive correlation between size of *P*. *forsteri* and coral colony size, although large individuals were still observed on small colonies and vice versa for all three species investigated, inferring size of coral colonies present does not have a strong effect on habitat preference of these hawkfish species. For *P*. *forsteri* this may be an artefact of the size range observed in this study (maximum size 13 cm TL) as this species reaches ca. 22 cm TL [32]. Additionally, location and the defence of food and sexual mates rather than shelter *per se* may explain the weak relationships between fish and colony size and the presence of individuals perching on colonies that appear too small for them to shelter within.

While the availability of preferred habitats (e.g., *Pocillopora* spp.) partially accounts for these large-scale differences in patterns of abundance, the local abundance and composition of hawkfish assemblages are likely to be structured by a range of other factors, including inter-specific competition, predation, prey availability and variation in larval supply and settlement of individual species. Many hawkfish species maintain a polygamous social structure with a single dominant male and several smaller subordinate females existing in non-overlapping territories [34,54,56]. While it has been hypothesised that territories are maintained to defend optimal microhabitats (e.g., coral colonies), recent evidence suggests that females defend food resources whereas the primary concern of males is defending territories against male conspecifics [54]. Substratum characteristics (e.g., coral cover, composition, complexity) may however still play a role in influencing territory size and some less dominant individuals may be forced to associate with, or occupy sub-optimal microhabitats. This may partially explain why hawkfish are not always observed on preferred habitat. In addition, environmental conditions (e.g., water quality, turbidity) and larval dispersal may also explain variations in distribution and abundance of hawkfish that are not fully explained by differences in benthic cover and composition.

The preferred hawkfish habitats, *Pocillopora* and *Acropora* colonies are highly susceptible to both physical and biological disturbances. Acroporid and pocilloporid corals are directly targeted by coral feeding starfish [57]; are vulnerable to climate induced coral bleaching [58] disease [59]; and their morphology renders them particularly susceptible to destruction from tropical storms [60]. In addition to reductions and degradation of available habitats, the loss of potential prey that is associated with these habitat types is expected to have a negative impact on these fishes. This study shows that hawkfishes displayed some flexibility in habitat use, suggesting they may not be significantly affected by patchy disturbances, although comprehensive and extensive coral loss is expected to have a detrimental affect on the abundance of the coral-dwelling species. For example, Pratchett et al. [22] documented that *P*. *forsteri* experienced significant declines in abundance following coral bleaching which resulted in greater than 50% coral loss. Interestingly, the same study found that *C*. *oxycephalus* increased in abundance following coral loss, suggesting habitat versatility is an advantage under a low-moderate coral loss scenario. If coral-dependent species of hawkfishes are impacted by environmental disturbances, there may be broader trophic consequences. Hawkfishes are important predators of small fishes and invertebrates on the reef as well as possible prey for larger fishes. Therefore, the loss or decline of these species may have far reaching effects for coral reef food webs.

This study illustrates the importance of live coral habitats for a small reef predator and builds on the growing list of fishes that have some dependency on live coral as habitat. The majority of these species appear to be habitat generalists but show strong selectivity for specific coral habitats when available (namely Acropora and Pocilloporidae). While the reasons behind the tight association between some hawkfishes and coral identity remains unresolved, it is likely that healthy branching live coral habitat provides important shelter from larger predators (e.g., [61]) as well as supporting the abundance and diversity of prey within their territories [49]. Investigating the specific habitat use and selection of reef fishes enables us to better appreciate how disturbances will impact fish communities and understand the flow on consequences for reef ecosystems. For some hawkfish species, their apparent preference for highly susceptible coral species may not be a successful strategy given the decline in coral cover at many locations (e.g., [27,29,62]) but their flexibility in habitat use may assist them following partial coral loss. For species that appear not to associate or select live coral habitats, the loss of live coral may have indirect effects, although further work on the effects of habitat disturbance on cryptic species is still required [15]. Additionally, it appears that for all these species, processes other than coral cover and composition also contribute to differences in abundances and distribution and drive patterns across geographic regions.

## Supporting Information

S1 Fig Fish size and habitat association. Mean size (total length ± SE) of each hawkfish species found associating with Pocilloporidae and *Acropora* species, and other hard coral species.
(EPS)

S2 Fig Relationship between fish length and coral size. Linear regression of fish length (total length cm) and coral size (max dia. cm) for three species of hawkfish.
(EPS)
